# Genetic Correction of Sickle Cell Anemia and β-Thalassemia: Progress and New Perspective

**DOI:** 10.1100/tsw.2010.67

**Published:** 2010-04-13

**Authors:** Ajay Perumbeti, Punam Malik

**Affiliations:** Divisions of Hematology-Oncology and Experimental Hematology/Cancer Biology, Cancer and Blood Institute, Cincinnati Children's Research Foundation, Cincinnati Children's Hospital Medical Center (CCHMC), Cincinnati, OH, USA

**Keywords:** gene therapy, hemoglobinopathies, sickle cell anemia, sickle cell disease, β-thalassemia, lentivirus, retrovirus, hematopoietic stem cells, insertional mutagenesis, β-globin, γ-globin, embryonic stem cells, inducible pluripotent stem cells

## Abstract

Gene therapy for β-globinopathies, particularly β-thalassemia and sickle cell anemia, holds promise for the future as a definitive corrective approach for these common and debilitating disorders. Correction of the β-globinopathies using lentivirus vectors carrying the β- or γ-globin genes and elements of the locus control region has now been well established in murine models, and an understanding of "what is required to cure these diseases" has been developed in the first decade of the 21st century. A clinical trial using one such vector has been initiated in France with intriguing results, while other trials are under development. Vector improvements to enhance the safety and efficiency of lentivirus vectors are being explored, while new strategies, including homologous recombination in induced pluripotent cells, for correction of sickle cell anemia have shown proof-of-concept *in vitro*. Here, a review is provided of the current substantial progress in genetic correction of β-globin disorders.

## INTRODUCTION

Inherited β-globin disorders (β-globinopathies), such as thalassemia and sickle cell anemia, are the most common monogenic disorders worldwide, and cause significant morbidity, mortality, and health care expenditures[1]. A one-time genetic correction has long been conceived as an ultimate method of cure. Current gene therapy technology has resulted in a permanent correction of other monogenic disorders, such as X-linked severe combined immune deficiency (X-SCID), adenine deaminase deficient SCID (ADA-SCID), and chronic granulomatous disease (CGD)[2,3,4,5]. In immunodeficiency diseases, relatively small proportions of gene-modified hematopoietic stem cells (HSCs) can give rise to sufficient long-lived T cells, and a relatively small amount of transgene protein expression in the differentiated T-cell progeny is adequate to produce a phenotypic correction. In β-globinopathies, requirements for effective correction of the clinical phenotype are much greater. An enormous amount of gene expression in individual red blood cells (RBCs), especially in RBCs from patients with thalassemia (Cooley's anemia), is necessary; additionally, engraftment of a large proportion of gene-corrected HSCs that repopulate the bone marrow is necessary in order to affect significant clinical effects[6]. HIV-1–based lentivirus (LV) vectors currently offer potential for delivering high expression and transducing a high proportion of HSCs, and have revolutionized genetic therapy approaches for β-globinopathies.

## HIV-BASED LENTIVIRUS VECTORS

Efficient delivery of large locus control region (LCR) elements and β-globin genes has been a challenging task with the conventional retroviral (RV) vectors. Numerous investigators have worked on defining critical elements within the LCR that are necessary for the high level of expression of human β-globin.

LV vectors:

1. Carry some unique *cis* elements that allow efficient packaging of the intron-containing β-globin gene expression cassette, allow stable transmission of the β-globin gene expression cassette, and allow stable transmission of the β- or γ-globin genes and the LCR.
2. Can carry larger cargo, allowing large LCR elements, necessary for high-level globin gene expression, to be inserted.
3. Can enter through the intact nuclear membrane of nondividing cells and integrate into nondividing HSCs at high efficiency. This eliminates the need for prolonged *ex vivo* cytokine prestimulation to force HSC division, a phenomenon shown to result in significant loss of "stemness".

Self-inactivating (SIN) LV vectors:

4. Are safer, as they carry no viral transcriptional elements and can be efficiently concentrated without significant loss in titers, a feature important for globin vectors that are typically produced at very low titers.
5. Are relatively resistant to proviral silencing due to lack of viral transcriptional elements[6,8,9], a phenomenon that has been observed commonly with conventional RV vectors in animal models[10] and in a recent clinical trial for CGD[11].

## GENE THERAPY FOR β-THALASSEMIA

Potential strategies for gene therapy of β-thalassemia have employed "additive gene therapy", delivering the human β-globin gene (hβ), recombinant hβ genes, or human γ-globin (hγ) genes, and the β-globin LCR enhancer into the HSC genome. Correction of thalassemic mice was first shown by May et al.[12] who delivered the hβ transgene via a LV vector. Mice that have heterozygous deletion of the mouse $\beta^{major}$ and $\beta^{minor}$ globin genes on one allele are commonly utilized as a model of thalassemia[13], and have a phenotype that resembles thalassemia intermedia with moderate anemia (hemoglobin [Hb] 8–10 gm/dl), allowing survival to adulthood. Homozygous deletion of the murine β-globin genes is lethal at embryonic days 14.5–15 due to a switch from embryonic mouse globin to adult $\beta^{major}$ and $\beta^{minor}$ in the fetal liver. When the fetal liver cells from E14.5 embryos are transplanted into lethally irradiated normal mice, the mice engraft with fetal liver HSC and develop severe anemia (Hb of 2–3 g/dl), like humans with Cooley's anemia, and die within 6 weeks. Genetic correction of fetal liver HSCs of thalassemia major mice by the Sadelain group[7], utilized the same hβ vector that corrected the thalassemia intermedia phenotype. Gene correction rescued the lethal phenotype and increased the hemoglobin by an average of 4 g/dl. Notable in their experiments was the variable correction among the different mice, showing that position effects play a significant role in therapeutic correction of the Cooley's anemia phenotype[7].

Subsequently, several groups have demonstrated correction in the thalassemia intermedia mouse model. Imren et al. showed correction of thalassemia intermedia using a LV vector with a mutated hβ (hβ$^{T87Q}$) that also had antisickling properties[14]. Emery et al. used a hγ and the smaller α-globin enhancer (HS-40) in a RV vector along with the chicken hypersensitivity site (cHS4) insulator element, and showed 5–45% hγ-expressing RBCs in the thalassemia intermedia mouse model[15]. Persons and colleagues showed that a similar level of expression of a γ-globin transgene delivered by a LV vector corrected the thalassemia intermedia phenotype in mice, but expression was dependent on the site of vector integration and copy number[16]. Taken together, these studies underscore the variable expression due to position effects, despite inclusion of large LCR elements.

Our group showed the first evidence of effective genetic correction of human Cooley's anemia using an *in vitro* and an *in vivo* xenograft model of human erythropoiesis with a cHS4-insulated β-globin LV vector, BG-I. The BG-I vector was used to transduce CD34+ cells from four patients with thalassemia major, of which three of the four patients had $\beta^0$ thalassemia major[17]. There was reversal of ineffective erythropoiesis and effective RBC production. Genetically modified thalassemia major CD34+ cells were then transplanted into splenectomized β2M$^{null}$-NOD-SCID mice and 3–4 months later, effective production of human glycophorin A–expressing erythroid cells were observed in circulation[17]. Miccio et al. showed that the hβ gene with a minimal 2.7-Kb HS2 and HS3 fragment could produce efficacious amounts of hβ protein. Importantly, the group demonstrated a selective advantage to erythroid progenitors and their expansion in mice[18].

In attempts to improve expression enough to correct β-thalassemia, Lisowski and Sadelain showed that the addition of HS1 of the LCR in the LV vector did not improve hβ expression, but the addition of HS4 in addition to HS2 and HS3 improved the expression from 27 to 41% per vector copy[19]. Hanawa et al. showed that use of a larger LCR fragment could improve overall expression of the hγ protein[20].

Our group subsequently showed that the cHS4 insulator element flanking hβ/LCR contributes to reduced variability of transgene expression, thus protecting the provirus from chromosomal position effects, and resulting in an overall two- to fourfold higher expression[8]. The uniform and, therefore, overall higher expression from insulated vectors corrects the human Cooley's anemia phenotype[17].

The $\beta^{87}$ LV vector developed by Leboulch and colleagues is now in a clinical trial in France for the treatment of sickle cell anemia and thalassemia. One patient with hemoglobin E-$\beta^0$ thalassemia transplanted with h$\beta^{87}$-modified CD34+ hematopoietic cells has become transfusion independent[21]. Of note, this transfusion dependence has occurred at the expense of a dominant integrant that is contributing approximately 85% of the $\beta^{87}$ globin. The LV is integrated in the HMGA2 gene intron in this clone, resulting in dysregulation of HMGA2, although the expression of $\beta^{87}$ globin is unaltered. So far, this "clonal dominance" seen at a molecular level has not caused any adverse event and has been beneficial to the patient[21]. (See Table 1 for a summary of recent gene therapy approaches for β-globinopathies.)

Of the newer technologies in development, Ye et al. produced human iPS cells from skin fibroblasts of patients with thalassemia major, and concomitantly showed that human iPS cells could be also be made from amniotic fluid cells and chorionic villus cells sampled for prenatal diagnostic for genetic and chromosomal disorders in the fetus[22]. This proof-of-principle study opens the door for possible prenatal production of iPS cells as a source for autologous HSCs for gene therapy and transplantation in the early neonatal period.

## GENE THERAPY FOR SICKLE CELL DISEASE

The strategy for gene therapy for sickle cell disease (SCD) is more complex, since it relies on production of an antipolymerization globin in a cell producing a full complement of globin chains. The well-known natural models for this antipolymerization effect are that patients with SCD are typically asymptomatic in the first 6 months of life when circulating sickle RBCs have high amounts of fetal hemoglobin (Hb F), and patients with SCD and hereditary persistence of Hb F have amelioration of disease. Both the naturally

**TABLE 1**
**Genetic Correction of β-Globinopathies**

| Vector | Cell Source | Model | Ref. |
|---|---|---|---|
| **β-Thalassemia** | | | |
| 1. h$\beta^{A}$ Lentivirus | Unselected bone marrow | Hb$\beta^{th3/+}$ TI | [12] |
| 2. h$\beta^{A}$ Lentivirus | 5-FU–treated bone marrow | C57BL/6Hb$\beta^{th-1}$/Hb$\beta^{th-1}$ | [14] |
| 3. hγ Lentivirus | 5-FU–treated bone marrow | Het KO $\beta^{major}$/KO $\beta^{minor}$ | [16] |
| 4. hγ Lentivirus | 5-FU–treated bone marrow | β-Thalassemia model | [20] |
| 5. h$\beta^{A}$ Lentivirus | Human thalassemia CD34+ cells | *In vitro* and nod/scid | [17] |
| 6. hγ γ-Retrovirus | 5-FU–treated bone marrow and D15.5 Fet. Liv. | Hb$\beta^{th-}$ and Hbb$^{th-3/th-3}$ | [23] |
| 7. h$\beta^{A}$ Lentivirus | 5-FU–treated bone marrow | Hb$\beta^{th+}$ | [19] |
| 8. h$\beta^{A}$ Lentivirus | Lineage, bone marrow cells | Hb$\beta^{th-}$ | [8] |
| 9. h$\beta^{A}$ Lentivirus | Untreated bone marrow | Hb$\beta^{th3+}$ | [18] |
| **Sickle Cell Anemia** | | | |
| 10. h$\beta^{AS3}$ Lentivirus | 5-FU–treated bone marrow | Berkeley + SAD | [24] |
| 11. h$\beta^{A-T87Q}$ Lentivirus | Lin$^{-(low)}$Sca-1$^{+}$c-Kit$^{+}$ cells | KO-TG sickle mouse | [25] |
| 12. h$\beta^{\alpha\ CMV}$ Cre Plasmid | eSC | Knock-in sickle mouse | [26] |
| 13. h -383 $\beta^{A}$ | iPSC | Knock-in sickle mouse | [27] |
| 14. hγ Lentivirus | Lin$^{-(low)}$Sca-1$^{+}$c-Kit$^{+}$ cells | Berkeley | [6] |

occurring Hb F and modified antisickling β-globins have been studied in the context of gene therapy for SCD.

Humanized murine models that result in murine RBCs with human hemoglobin, which have been used for this purpose, are the Berkeley sickle mice developed by Paszty et al.[28] and the knock-out transgenic (KO-TG) sickle mice developed by Townes and colleagues[25,26]. Gene transfer experiments are performed by transplanting gene-modified murine sickle stem/progenitor cells into lethally irradiated normal mice, since sickle mice do not tolerate myeloablative irradiation. Transplant of unmodified HSCs (mock controls) would convert a normal mouse to a sickle mouse following engraftment, while genetic correction would correct the SCD phenotype.

To circumvent the problem of low hγ expression in adult/postnatal RBCs, synthetic antisickling β-globins ($\beta^{T87Q}$, $\beta^{AS3}$) were designed by the Leboulch and Townes groups, respectively, which prevent contacts between β-sickle ($\beta^{S}$) globin and α-globin, thus preventing polymer formation[29,30,31]. Pawliuk et al. showed correction of humanized Berkeley sickle mice with delivery of the $\beta^{T87Q}$ LV vector[24]. Levasseur et al. showed correction of the KO-TG sickle mice using a highly HSC-enriched population [Lineage(-) Sca(+), c-Kit(+) cells] and showed that expression of 20–25% h$\beta^{AS3}$ could completely correct SCD in primary and secondary mice[25]. The h$\beta^{AS3}$ globin carries three mutations and is functionally more potent than the $\beta^{T87Q}$ globin; and as potent as hγ in inhibiting sickle polymer formation. In an attempt to reduce h$\beta^{S}$ concomitantly, Samakoglu et al. expressed hγ with a small hairpin RNA targeting h$\beta^{S}$ mRNA via a LV. There was reduction in h$\beta^{S}$ mRNA along with increased expression of hγ mRNA in human CD34+ cell erythroid progeny[32]. This approach was effective, although the findings need confirmation with protein expression and *in vivo* models, the latter necessary to assess the toxicity of shRNA vectors. Subsequently, our group[6] and that of Persons[33] showed correction of the Berkeley sickle mouse with a hγ-globin vector alone. (See Table 1 for summary of preclinical gene therapy studies in β-globinopathies.)

## REQUIREMENTS FOR SUCCESSFUL GENETIC CORRECTION

While the above approaches have yielded elegant proof-of-concept data, major concerns for current models have been (1) utilizing a “disease prevention” model, attained by transplantation of genetically corrected or uncorrected progenitors into myeloablated “normal” mice; (2) lack of disease sequelae/organ pathology in recipient mice; and (3) complete donor chimerism with myeloablative regiments results in a high degree of chimerism in gene-corrected HSC in mice, findings that are not practically achieved in humans. Indeed, it has taken 2 decades of multiple clinical trials in ADA-SCID before critical determinants for successful correction were defined. Therefore, there is a strong need for assessing requirements for successful correction of the disease in humans, following carefully designed animal models, after initial proof-of-concept studies.

To examine the minimal proportion of gene-corrected long-term repopulating cells necessary to cause repopulation of genetically corrected RBCs and ameliorate the SCD phenotype, we transplanted varying doses of gene-transduced Berkeley sickle HSCs into Berkeley sickle recipients exposed to nonmyeloablative conditioning. We have utilized a modified hβ LV vector, where the exons of hβ are replaced by hγ exons, but the introns, promoter, and 3’ and 5’ untranslated regions are those of the hβ gene. A careful quantification of the hematological correction, functional correction (degree of sickling, RBC half-life, and deformability), and correction of organ pathology showed that when genetically modified HSCs result in 20% or more of the repopulated donor marrow, the total Hb F produced is greater than 10%, with at least 60% Hb F containing RBCs (F cells) and Hb F per F cell represents 30% of the total globin in the RBCs. This results in an effective and successful phenotypic correction of SCD in the Berkeley mice[6] (illustrated in Fig. 1). More importantly, we could achieve this with nonmyeloablative conditioning in this model, suggesting the same could be tried in human trials initially, which could significantly reduce potential morbidity of this approach.

New modalities that are currently being investigated are the gene correction approach with homologous recombination[26]. Hanna and colleagues[27] generated iPS cells from the “knock-in” mouse tail fibroblasts, performed homologous recombination, selected corrected clones, and differentiated them into HSCs *in vitro* via gene transfer of the HoxB4 transcription factor. HSCs developed from murine iPS cells were transplanted into lethally irradiated normal mice and resulted in normal erythropoiesis and correction of SCD[20]. The pluripotency and growth potential of iPS cells can potentially overcome inefficient gene delivery by homologous recombination, by selecting genetically corrected clones confirmed by molecular analysis and expanding them. Since iPS cells are pluripotent cells derived from mature cells, and not embryos, this may be more acceptable to those concerned about the use of embryos to generate cellular therapies. Human ES cells, differentiated *in vitro* into HSCs, only give rise to erythroid cells producing embryonic and fetal globins[34]. Proof-of-concept studies on human iPS cells and their differentiation into HSCs has been shown *in vitro*, but these cells are not transplantable[35]. Transplantable HSCs that contribute to definitive erythropoiesis are needed and are under active investigation. These approaches are novel and exciting, but are farther away from translation into the clinic, and much work needs to be done to show their expansion capability, long-term safety, and feasibility for human use.

## SAFETY OF LV VECTORS FOR β-GLOBINOPATHIES

Safety of gene therapy is of paramount importance for additive gene therapy with randomly integrating vectors. Of particular concern are gains of function in the form of (1) insertional activation of oncogenes or (2) dysregulation of gene expression resulting in increased/unregulated target cell proliferation. Loss of function resulting from disruption of a cellular tumor suppressor gene that results in loss of heterozygosity could also potentially occur. The potential for leukemogenesis with gene therapy has been shown to be more than a hypothetical risk by the development of T-cell leukemia in five of 20 patients in

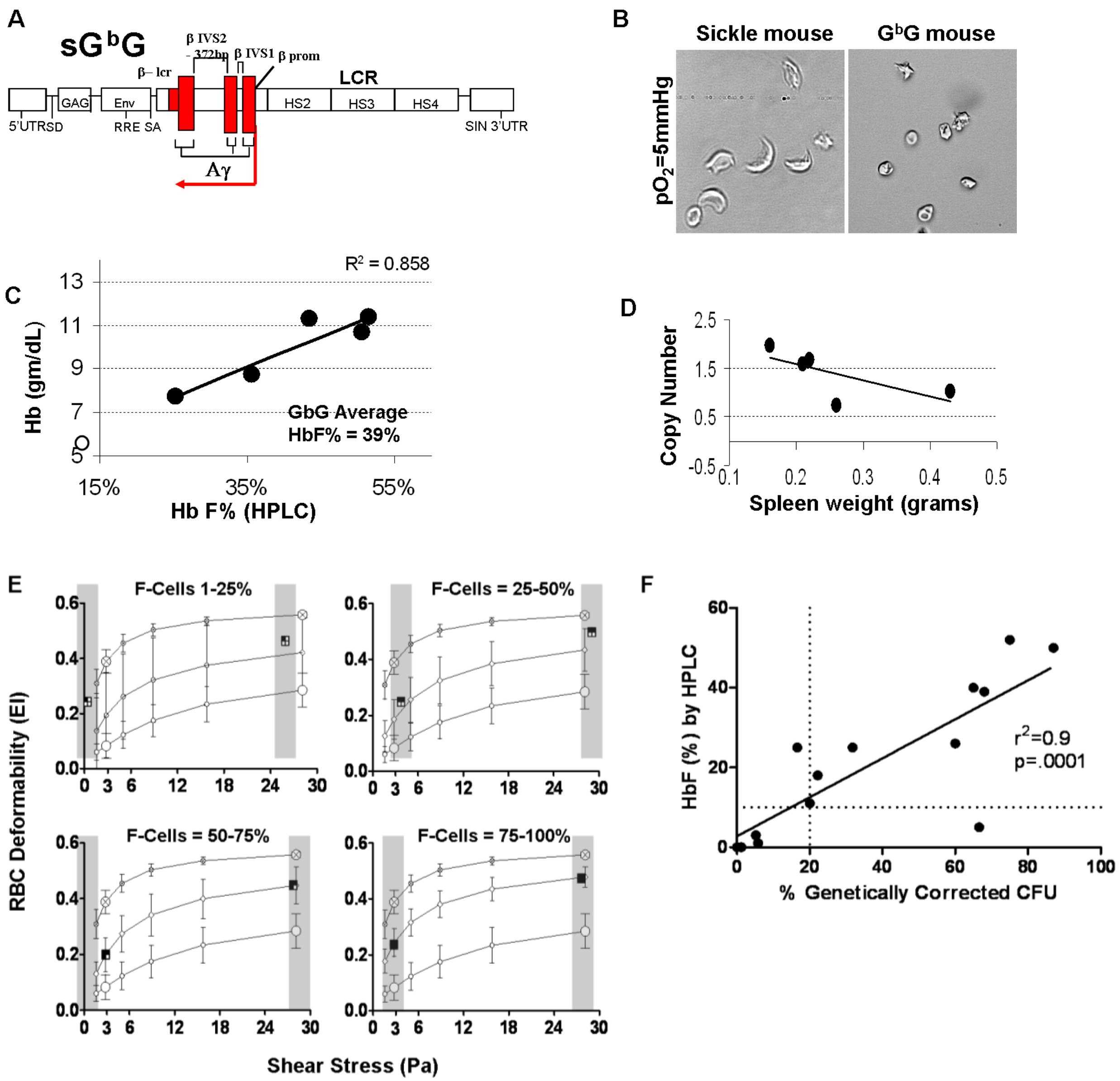

**FIGURE 1.** Critical determinants required for correction of SCD in Berkeley sickle mice. (A) sGbG vector encodes Aγ-globin exons, β-globin promoter, and introns, and HS2, HS3, and HS4 elements of the LCR. (B) GbG mice (sickle mice transplanted with $sG^bG$ genetically modified HSCs) RBCs are more resistant to hypoxia-induced sickling than "Mock" transplanted sickle mice. (C) Increasing hemoglobin with increases in Hb F expression. (D) Reduction of extramedullary hematopoiesis as demonstrated by reduced spleen size with increasing copies of transgene. (E) Peripheral blood from sickle mice was exposed to shear stress and RBC deformability was measured using laser-assisted optical rotational cell analyzer (LORCA). GbG mice showed significant improvements in RBC deformability at low and high shear pressures as F cells increased. (F) Colony forming unit spleen (CFU-S) assay was performed on GbG mice 6–11.5 months following transplant, showing that when the proportion of GbG-modified HSCs were roughly 18–20%, the percentage of Hb F was greater than 10%. $r^2$ reflects correlation excluding single outlier. Overall, greater than 18% genetically transduced HSCs correlates with improved hematological and functional parameters of SCD and improved survival (data not shown).

the French and British human gene therapy clinical trials performed for X-SCID, and development of myelodysplastic syndrome in two of two subjects in the clinical gene transfer trial for CGD using γ-RV vectors[5]. In both trials, there was insertional activation of surrounding cellular proto-oncogenes by the viral long-terminal repeat (LTR) enhancer. Strangely, no such predilection was observed in the gene transfer trial for ADA-SCID performed using a similar RV vector with long follow-up[2]. The adverse events from the X-SCID and CGD trials have prompted deeper investigations into vector genotoxicity and have shown that presence of ubiquitous enhancers in viral vectors are not desirable. Experimentally, SIN LV and RV vectors containing internal promoters/enhancers show reduced genotoxicity, although strong ubiquitously active enhancers can dysregulate genes independent of vector type/design[36].

## Genotoxicity of the Locus Control Region in Globin Vectors

Imren et al. showed integration of LCR-containing hβ LV vector near putative oncogenes in primary human cells[37], although expression of the proto-oncogenes was not analyzed in their study. Recently, some global perturbation of gene expression in colony forming units spleen (CFU-S) with the LCR-driven LV was reported in microarray analysis[38]. It was also reported that the cHS4 insulator in RV results in reduced perturbation of gene expression in microarray analysis[39].

The LCR is a potent long-range lineage-specific enhancer that has been shown to be in an active chromatin configuration prior to erythroid lineage commitment in multipotent HSCs[40]. We therefore explored the genotoxicity of β-globin LCR with/without insulator elements using primary murine hematopoietic progenitors to generate *in vitro* immortalization assay mutant clones[41]. LCR-containing LV vectors had approximately 200-fold lower transforming potential when compared to the conventional RV vectors with intact LTRs. The LCR perturbed expression of a few genes in a 300-Kb proviral vicinity, although no up-regulation of genes associated with cancer, including an erythroid-specific transcription factor, occurred. A further two- to threefold reduction in transforming activity was observed with insulated LCR-containing LV vectors. It should be noted that mock transduced lineage-negative progenitors, and those transduced with vectors carrying the hβ promoter, but no LCR, did not transform any lineage-negative progenitors; these studies clearly demonstrated that the low level of transforming potential was due to the LCR enhancer, and also showed the robustness and sensitivity of the *in vitro* immortalization assay. *In vivo* models for vector-induced leukemias at best generate tumors in one in ten mice when studied long term in secondary transplants. Our data suggest that thousands of mice would be required to demonstrate genotoxicity *in vivo*[41].

## Successful Early Gene Therapy Trial in β-Thalassemia

Preliminary results from the first trial of gene therapy for thalassemia and SCD by Leboulch and colleagues were presented at the American Society of Gene Therapy Annual Meeting 2009[21]. One patient with hemoglobin E-$\beta^0$ thalassemia was treated with genetically modified CD34+ cells (using the $h\beta^{T87Q}$ LV vector) in June of 2007 and has demonstrated persistence of long-term gene-marked cells, and the “holy grail” of therapeutic benefit in becoming RBC transfusion independent. Integration analysis revealed that a dominant clone with integration within the HMGA2 gene was detected in myeloid and erythroid cells, but not lymphoid cells (NIH Office of Biotechnology Activities, News and Events, Recombinant DNA news, June 15, 2009, based on a press release by French Health Products Safety Agency [Agence Française de Sécurité Sanitaire des Produits de Santé; AFSSAPS]). The HMGA2 gene expression is increased in the erythroid cells, but not the myeloid cells, showing dysregulation of this gene by the LCR in the erythroid lineage. The transplant of transduced CD34+ cells, which included myeloablation, has resulted in a transient net increase in the patient’s Hb F, and longer, persistent expression of the vector-derived $\beta^{87}$ globin, resulting in transfusion independence. Therefore, this insertional dysregulation has resulted in a clinical benefit and clinical phenotypic correction of the anemia, the predominant disease pathology of thalassemia. However, this is also the first clinical evidence of the emergence of a dominant cell population in a human clinical trial with LV vectors, and whether the clinically beneficial effect of this clonal dominance continues long-term remains to be seen. The AFSSAPS has allowed the trial to accrue more patients[21].

## IMPROVING EFFICIENCY OF GLOBIN VECTORS WITH CHROMATIN INSULATORS

Silencing and position effects, due to integration of viral vectors in heterochromatin regions, are significant obstacles to obtaining consistent or uniform levels of transgene expression. Silencing of transgenes has been shown *in vitro* and in murine models[42,43,44] and, recently, in two patients treated for CGD[5], where silencing of the vector promoter abolished the therapeutic effect[11]. We have not observed silencing with our vector or the LCR/β-promoter SIN LV vector backbone in mice. Nevertheless, shielding the vector with a chromatin insulator could achieve consistent expression of γ-globin independent of integration sites, resulting in similar Hb A or Hb F levels at much lower vector copies, thereby improving efficiency and safety[8,45].

Inadvertent activation of surrounding cellular proto-oncogenes by viral enhancers has led to leukemia and myelodysplastic syndrome in three gene therapy trials[5,46,47,48], underscoring the need for enhancer-blocking elements. While current globin LV vectors have several features that impart safety (i.e., a self-inactivating design, erythroid-specific expression, and generation of enucleated progeny that cannot be transformed), the LCR enhancer can dysregulate genes to a small extent, as shown by us and others[21,38,41]. Efficient insulators can block the LCR enhancer from dysregulating genes surrounding the provirus and further improve the safety of the vector, as shown by the cHS4 insulator[8,49,50].

Only a limited number of chromatin insulators have been identified and/or characterized. One of the best-characterized insulators, cHS4, has enhancer-blocking activity[49,51,52) and resists proviral silencing to provide uniform expression independent of the integration site[8,15]. For LV vectors flanked by cHS4, we demonstrated remarkable protection from position effects, fourfold higher globin protein[8], and two- to threefold improved safety[41]. In order to flank the transgene expression cassette, insulators need to be placed in the 3’ LTR, since repeat elements within RV vectors are unstable. However, LV vectors carrying globin genes have modest transduction titers, and the large size of cHS4 (1.2 Kb) in the 3’ LTR reduces reverse transcription and integration efficiency, causing further severe reductions (10- to 15-fold) in titers[53], thereby precluding clinical-scale vector production.

The insulator activity of cHS4 has been mapped to a 5’ 250-bp sequence (the “core”), where CTCF and USF-1/2 elements impart enhancer-blocking[49,51,52] and PE[8,15] activity, respectively. Although insertion of the core in the LTR minimally reduces titers[53], we show that it is ineffective at insulating LV[45]. Indeed, Leboulch and colleagues demonstrated failure of the cHS4 core in the one patient receiving cells transduced with $\beta^{87}$ globin LV vector[21], where HMGA2 expression was greatly enhanced in erythroid cells by the LCR and led to erythroid proliferation, despite the cHS4 core in the provirus[21]. While this proliferation turned out to be beneficial, the potential of this occurring in the context of an oncogene is worrisome.

We recently mapped a unique insulator activity to a 3’ 400-bp sequence of the cHS4 element. This region has no known CTCF/USF-1 sites, but recruits CTCF on its own and USF-1 from the canonical 5’ core (Fig. 2). The interaction of the 3’ 400-bp sequence and the 5’ 250-bp core of cHS4 is necessary for active chromatin configuration of the transgene promoter, and cHS4 and full insulator activity. Therefore, the 3’ 400-bp region has critical elements that complement the core to impart full insulator activity. Vectors carrying the core and 3’ 400-bp (650-bp) sequences of cHS4 have only a modest (2.5-fold) reduction in titers, yet retain full insulator activity in MEL clones, in RBCs of thalassemia mice, and in secondary CFU-S[45]. Further studies on $sBG^{650}$ and sBG-I proviruses, showed that the 5’ core and the 3’ 400-bp sequences had increased active histone marks/acetylation (AcH3,AcH4 and H3K4-me3) and reduced repressive histone marks over the insulator and the β-globin gene promoter.

This effect was not seen with the core alone or extensions of the core encompassing up to 800 bp of 5’ cHS4 sequences[45]. The combination of proximal and distal ends of the cHS4 was necessary for open chromatin patterns. Our epigenetic analysis suggests that the 5’ and 3’ ends of the cHS4 insulator function as two “cores”, which must interact for optimal epigenetic modifications to impart adequate insulator activity. The LV vector carrying either the 650-bp or the 1.2-Kb cHS4 had lower *in vitro* genotoxicity than their uninsulated counterpart[41]. Identification of novel proteins that complement interactions of USF and CTCF will result in identification and/or design of more potent insulators.

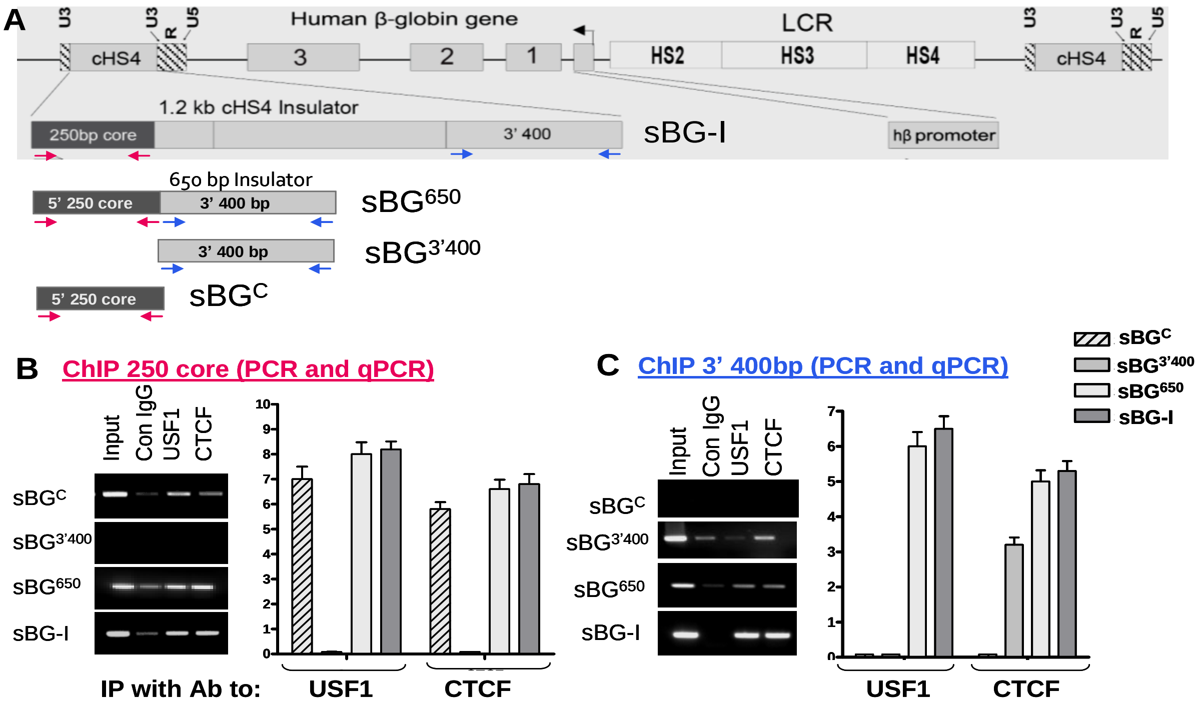

**FIGURE 2.** (A) Design of the SIN β-globin/LCR LV vector sBG with cHS4 inserts in the 3' LTR. Vectors with various insulator inserts are depicted with the organization of cHS4. The cHS4 core is present in the 5' 250-bp region. (B, C) ChIP for the 250-bp core and 3' 400-bp fragments with antibodies to USF-1 and CTCF followed by semi-quantitative PCR (gels) or qPCR (bar diagrams), on DNA sonicated to 200-bp fragments, from transduced pools and three single copy clones of MEL cells carrying the $sBG^{C}$, $sBG^{3'400}$, $sBG^{650}$, and sBG-I proviruses.

## SUMMARY

Gene therapy for Cooley's anemia and SCD offers promise as a treatment approach in the future. Correction and cure has been well established in murine models and human models of the disease with HIV-1–based LV vectors. Projections of what levels of gene expression are necessary to correct these diseases clinically have been realistically assessed in the first decade of the 21st century. The current limitations for genetic correction are vector production, efficient gene delivery into a high proportion of engraftable HSCs, and adequately high expression and persistence of the transferred gene, to a level that would be curative in the human setting. This may require only a relatively low proportion of corrected cells in the marrow. Long-term safety of the approach must be established in clinical trials. Currently, clinical trials are being developed by several groups that have performed correction in animal models of globin diseases, and a clinical trial is underway with highly promising results in abolishing previous transfusion dependence in HbE-β thalassemia in France by Leboulch and colleagues. As progress is made in these areas, gene therapy may indeed achieve the long-sought goal of ameliorating the most common monogenic diseases in the world.
